# Chronic Obstructive Pulmonary Disease Management in the Real World: The Importance of a Holistic Assessment

**DOI:** 10.7759/cureus.84006

**Published:** 2025-05-13

**Authors:** Ana Cristina Franco Spínola, Nivalda Pereira

**Affiliations:** 1 Family Medicine, Centro de Saúde do Bom Jesus, Serviço de Saúde da Região Autónoma da Madeira (SESARAM) Entidade Pública Empresarial Regional Autónoma da Madeira (EPERAM), Funchal, PRT; 2 Family Medicine, Centro de Saúde Dr. Rui Adriano de Freitas, Serviço de Saúde da Região Autónoma da Madeira (SESARAM) Entidade Pública Empresarial Regional Autónoma da Madeira (EPERAM), Funchal, PRT

**Keywords:** chronic obstructive pulmonary disease, health literacy, integrated healthcare, primary care, smoking cessation

## Abstract

Chronic obstructive pulmonary disease (COPD) is a prevalent condition in smokers that is often underdiagnosed. It offers significant challenges in daily primary healthcare practice as the disease needs a holistic approach. The wait times for appointments with the family doctor are longer than desired; its duration is short and inadequate for the complex and time-consuming approach to COPD; spirometry can only be performed in secondary healthcare services, limiting its assessment in a timely manner; and pulmonary rehabilitation is nonexistent in our primary healthcare context. These are some examples of the resource scarcity we face. In addition, the patient's low literacy towards COPD obstructs behavioural change and treatment compliance. Reporting a clinical case from our reality aims to improve our practice by reflecting on points to target next in a continual training and learning process within our teams. We present the case of a 55-year-old man with COPD type E, a current smoker, with severe exacerbations over the course of a year. These required a closer follow-up, with the involvement of more healthcare professionals, from different areas of expertise, and regular non-pharmacological therapeutic interventions. We discuss the challenges we have faced, from the patient's and healthcare team's perspective, towards an integrated approach.

## Introduction

Chronic obstructive pulmonary disease (COPD) is a heterogeneous lung condition characterized by chronic respiratory symptoms due to abnormalities of the airways and/or alveoli that cause persistent, and often progressive, airflow obstruction [1,2].

In the appropriate clinical context, FEV1/FVC<0.7 post-bronchodilation with a negative bronchodilation test, assessed by spirometry, confirms the diagnosis of COPD. Patients typically complain of dyspnea, activity limitation, and/or cough with or without sputum production and may experience acute respiratory events characterized by increased respiratory symptoms called exacerbations that require specific preventive and therapeutic measures [1,2].

Among the main environmental exposures leading to COPD is tobacco smoking. Approximately 40% of people with COPD are current smokers, and this behaviour has a negative impact on the prognosis and progression of the disease [1]. Smoking cessation is the most cost-effective measure to change COPD's natural course [1,2]. The specialized smoking cessation appointment is a structured program, carried out by trained healthcare professionals in a multidisciplinary setting, for smokers motivated to quit smoking, with a behavioural and pharmacological approach over 12 months of follow-up. If effective resources and time are dedicated to smoking cessation, long-term quit success rates of up to 25% can be achieved, with identical value in our in-house data.

In addition to quitting smoking, other lifestyle changes can help manage COPD; these include regular exercise, an appropriate diet, and avoiding exposure to environmental pollutants. Vaccinations for influenza, SARS-CoV-2 (COVID-19), pneumococcal disease, respiratory syncytial virus, pertussis, and herpes zoster are recommended for individuals with COPD [1].

Pulmonary rehabilitation is a non-pharmacological intervention based on thorough patient assessment followed by patient-tailored therapies that include exercise training, education, and self-management intervention. It is designed to improve the physical and psychological condition of people with COPD by improving dyspnea, overall health status, and tolerance to exercise and ameliorating symptoms of anxiety and depression. Additionally, it promotes the long-term adherence to health-enhancing behaviours [1].

Inhaled bronchodilators are the main pharmacological intervention in COPD, impacting pulmonary function, dyspnea, and overall health status and reducing exacerbations [1].

Individuals with COPD frequently present other chronic diseases. These impact the progression of the disease and the patient's quality of life, increasing the risk of hospitalization and death. Comorbidities such as anxiety, depression, and cognitive deficit are underdiagnosed, and a systematic approach to the patient is advised [1].

## Case presentation

**Figure 1:**
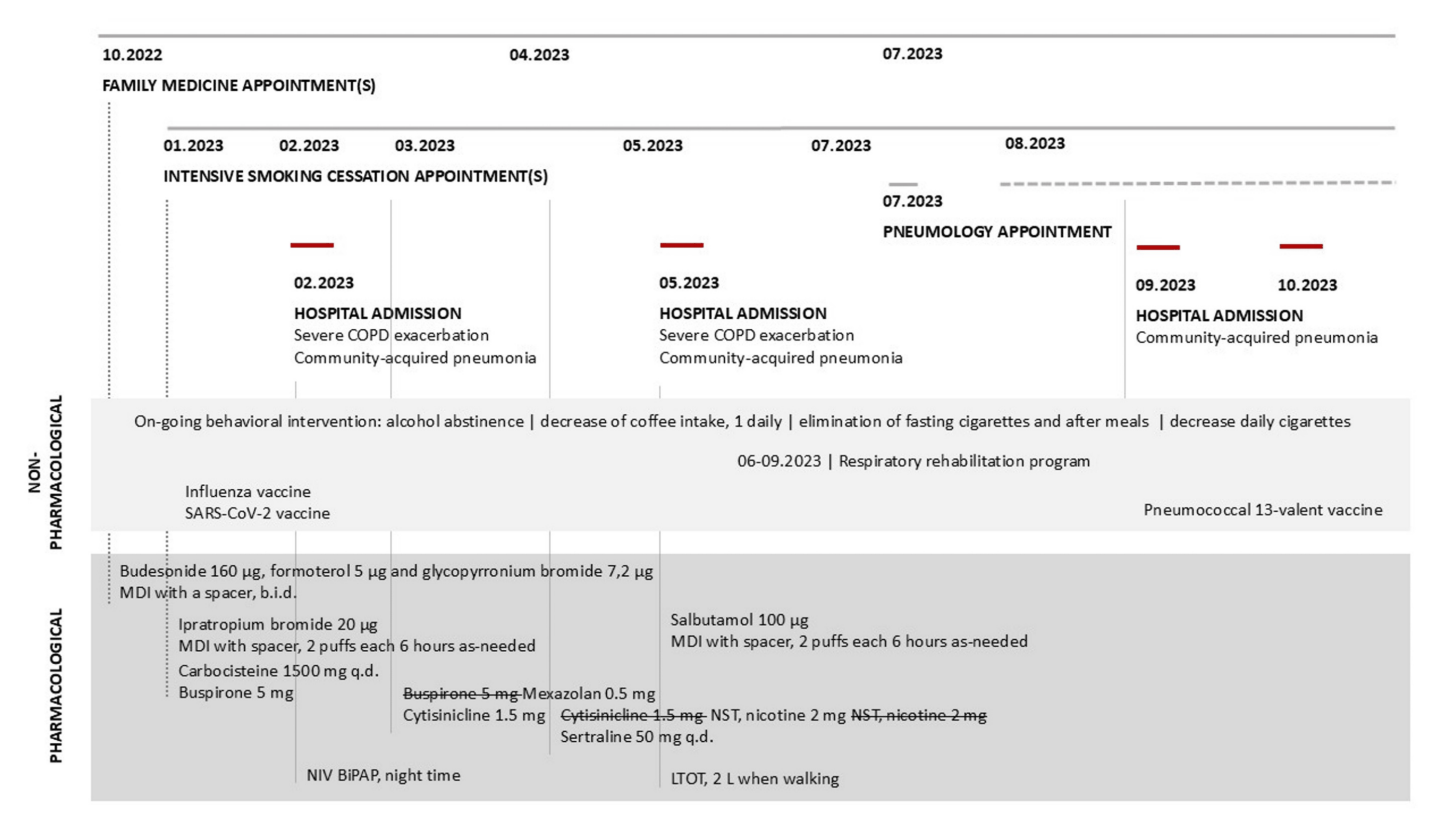
Appointments and non-pharmacological and pharmacological walkthrough COPD: chronic obstructive pulmonary disease; MDI: metered dose inhaler; NIV: non-invasive ventilation; LTOT: long-term oxygen therapy

A 55-year-old Portuguese man presented to his family doctor in October 2022 with dyspnea of more than 12 weeks of evolution. He is divorced and lives alone in a room, with a limited supportive network and no relation to his kin except to a sister, who accompanied him in seldom appointments. He completed six years of basic education, worked as a constructor until 2010, and has been unemployed since then. He receives social security support for food acquisition and meal preparation. He did not have any relevant medical history, namely, cardiovascular risk factors, prior myocardial infarction, nor stroke; he had a healthy childhood, no pneumonia in adult age, nor lung tuberculosis (or had been in contact with). He was not a regular primary healthcare service user and had no prescribed medication or known allergies. The national vaccination plan was updated. He smoked 45 pack-years for 45 years and consumed 7-9 standard drinks daily, twice to thrice a week. His body mass index was 16.79 kg/m^2^, and he reported a recent weight loss of 10 kg, due to low food intake. On physical examination, he was eupneic at rest (modified Medical Research Council (mMRC): 2), with a pulse oximetry of 92% on room air at sea level, with globally diminished lung sounds, bilateral wheezes, and rhonchus, that correlated to the cough, sputum production, and dyspnea that he reported. Given his smoking habits, he was referred to a smoking cessation consultation and to pneumology for the initial assessment and realization of a forced spirometry, as COPD was then presumed. Concomitantly, he was prescribed a combination of budesonide 160 µg, formoterol 5 µg, and glycopyrronium bromide 7.2 µg in a metered dose inhaler (MDI), with a spacer, b.i.d., as the previous blood eosinophil count was higher than 300 cells/μL (Figure 1). A follow-up appointment was scheduled within two weeks.

**Figure 2:**
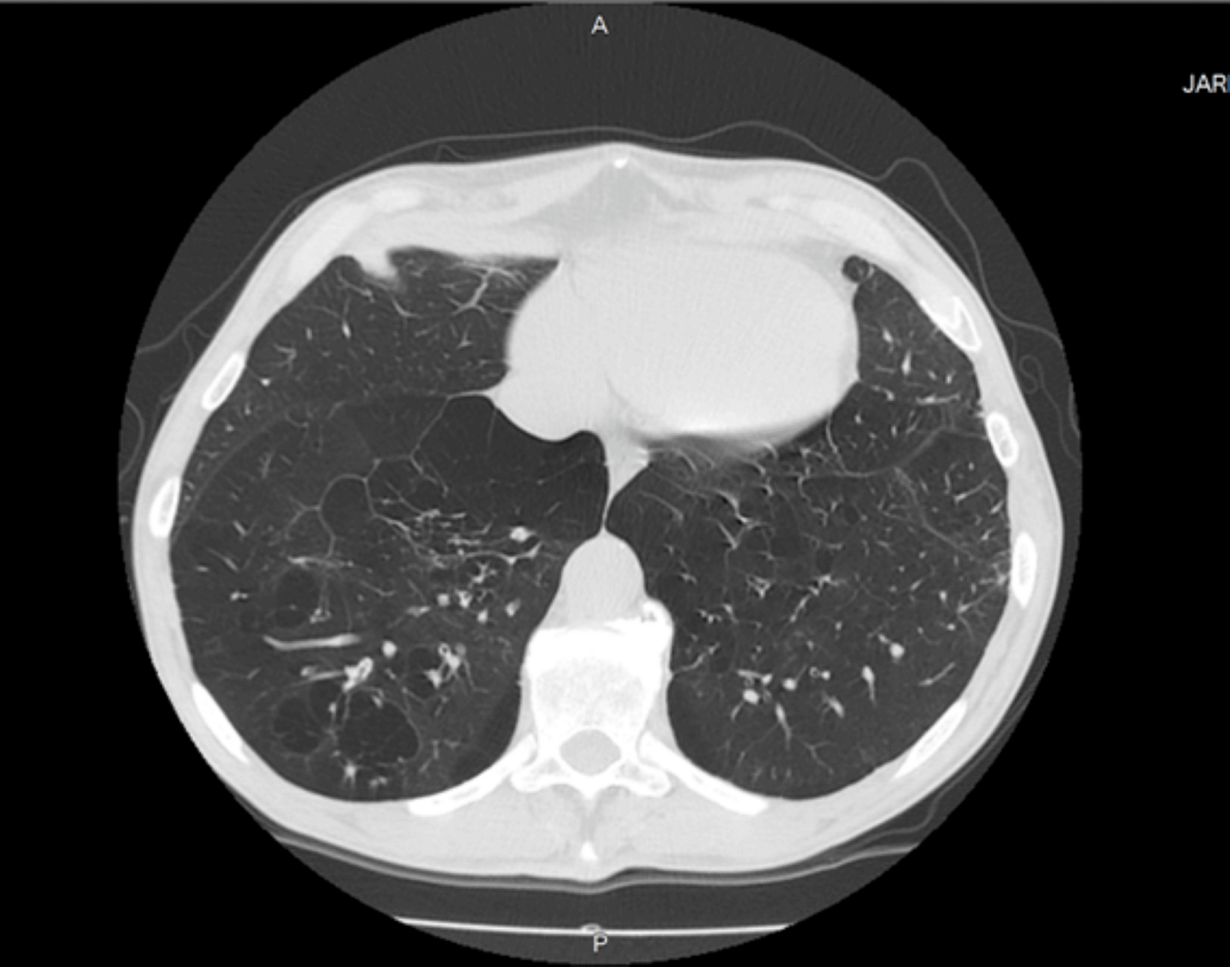
CT scan, at the lung basis' plane, November 2022 Centriacinar emphysema with dispersed and multiple blebs, with an exuberant superomedial, paraseptal, and cross-mediastinal major pleural bleb. Preserved airway caliber; discrete peribronchitis, with thickening of the inferior bronchi walls. Bronchiolectasis and small distal blebs outline trapped secretions, mostly in the right lung base; varicosed middle segmental bronchus. Preserved central airways, with dystrophic parietal calcifications. Pulmonary arteries with a diameter of 34 mm, suggesting pulmonary hypertension. Discrete mediastinal ganglia with nonspecific characteristics. No pleural or pericardial effusion.

**Figure 3:**
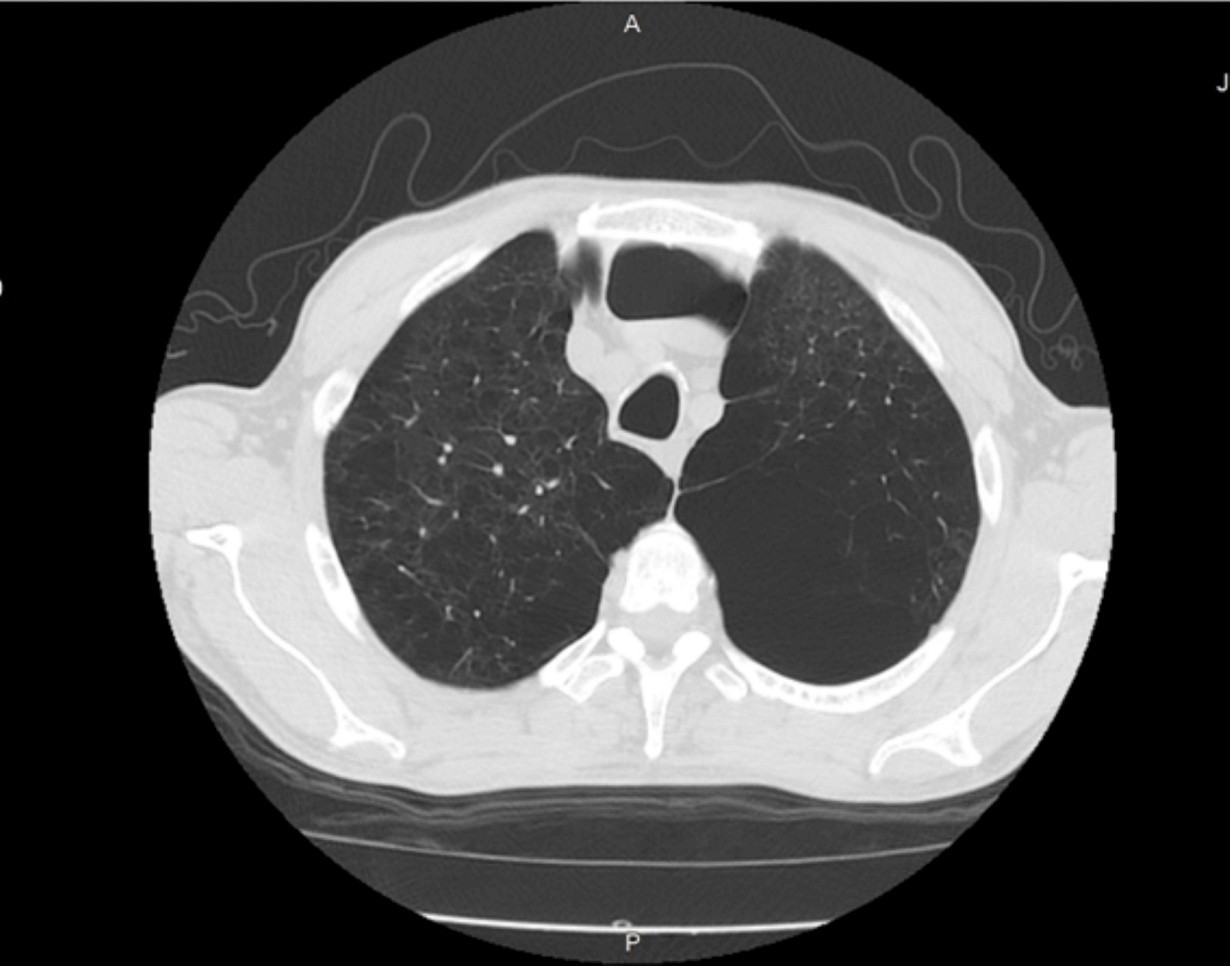
CT scan, at the mid-lung's plane, November 2022

Following a routine assessment, in November 2022, the patient was assisted in the emergency department due to low peripheral oxygen saturation (SpO2) of 86% on room air at sea level and aggravated dyspnea. A CT scan performed at that time (Figure 2 and Figure 3) documented a distorted lung architecture, with exuberant emphysema areas and multiple bronchiolectases outlining trapped secretions. 

In January 2023, at the time of the first smoking cessation appointment, in primary care, composed of a doctor and a nurse, he was mildly motivated to cease smoking (Richmond test score: 5 out of 10), expressed fear of non-accomplishment, and reported an aggravating dyspnea that worried him. Lifestyle intervention was targeted at decreasing alcohol and cigarette intake. In this appointment, behavioural changes were negotiated with the patient in a shared process, aiming to reduce coffee intake to one daily, out of the three he had together with a cigarette, as coffee increases smoking cravings, and to eliminate cigarettes with fasting and allowance of a maximum of 15 cigarettes per day. Influenza and COVID-19 vaccinations were performed that day, after a shared decision with the patient, and rules on cigarette use were reestablished, along with recommendations on decreasing alcohol intake. Carbocisteine 1500 mg q.d. was introduced in the regular therapeutic regimen to reduce sputum production, with ipratropium bromide 20 µg, MDI with spacer, two puffs every six hours when with dyspnea.Buspirone 5 mg was prescribed to ameliorate nicotine and alcohol withdrawal symptoms.

In February 2023, he was observed in an unscheduled appointment, due to agitation, increased dyspnea, polypnea, and cyanosis since that morning. He was apyretic, with a SpO2 of 42% on room air at sea level, normotensive, and tachycardic (108/65 mmHg, 136 bpm), with diminished vesicular murmur, abundant rhonchus, and global wheezing. The patient was then transferred to the nearby emergency department, in need of non-invasive ventilation (NIV), and hospitalized thereof with community-acquired pneumonia and global respiratory insufficiency with respiratory acidemia. He completed seven days of levofloxacin 750 mg q.d. and a short course of respiratory rehabilitation and was discharged with domiciliary NIV (bilevel positive airway pressure (BiPAP) at night).

In mid-March, at the time of the fourth follow-up smoking cessation consultation, he had decreased his cigarette consumption to 5-6 cigarettes a day and was abstinent from alcohol. He did not bring his medication but reported having stopped the budesonide 160 µg, formoterol 5 µg, and glycopyrronium bromide 7.2 µg and, instead, using the ipratropium bromide 20 µg and MDI with a spacer in a regular two puffs b.i.d. Medication reconciliation was then made, and instructions were given to bring all medication in use to the next appointment. Cytisinicline 1.5 mg was added to the regimen, and mexazolam 0.5 mg p.o. was prescribed as needed, instead of buspirone 5 mg. Following two weeks, the inhaler technique was assessed, and errors were corrected. An adapted written regimen for cytisinicline was prescribed, and a follow-up appointment was scheduled. Since the patient managed not to cease smoking with cytisinicline, this was stopped, and nicotine replacement therapy was initiated. However, no further results were achieved with this latter therapy. For these reasons, we ceased the pharmacological approach, switching the focus to the number of cigarettes allowed per day that we renegotiated with the patient. An underlying depression aggravated this context further, and for this reason, sertraline 50 mg q.d. was added to the therapeutic regimen in April 2023.

During the year, three other admissions to the hospital occurred: in May, due to a community-acquired pneumonia, where he completed seven days of piperacillin-tazobactam and was prescribed long-term oxygen therapy (LTOT), and in September and in October, because of aggravated cough, dyspnea, and peripheral desaturation, as a result of medication non-compliance and NIV misuse. Between June and September, he completed an intensive pulmonary rehabilitation program. The pneumococcal vaccination was initiated in September. Due to limitations in cost from the patient side, the Pneumococcal 13-valent conjugate vaccine, with an estimated market value of 30 euros, was preferred to the 20-valent's, with an estimated market value of 70 euros. In a shared decision with the patient, it was explained that the Pneumococcal 20-valent vaccine is more consistent with Portuguese epidemiology, protecting for a higher number of bacteria serotypes, and has a higher impact on morbidity and mortality by pneumonia. However, this latter vaccine was non-reimbursed at that time, in opposition to the 13- and 23-valent regimen. In August, the case was presented to the palliative care team, composed of a medical doctor and nurse, because of its complexity and increasing exacerbations, and an initial assessment was done at home by the palliative nurse assessing end-of-life objectives.

## Discussion

Smoking cessation is the most cost-effective measure to change COPD's natural course. Individual approaches are effective, and counselling delivered by healthcare professionals improves quit rates. The positive reinforcement for smoking cessation, periodical motivation assessment, and ongoing adjustment of the non-pharmacological and pharmacological strategies are pivotal. Integrated approaches to care are still necessary, as long-term smoking cessation rates remain low at 25% [3]. Beyond this, health policies need to be implemented to reduce smoking consumption.

Physical activity improves quality of life and reduces hospitalization and mortality in COPD patients [1]. However, social isolation, physical limitations, and mental constraints, as described in this case, can prevent this from becoming a reality.

A nutritional approach could have been made available for this patient throughout the progression of the disease. Despite being available in our setting, he was not motivated by this approach.

Vaccination updates were discussed in each encounter with the patient and prioritized thereof, firstly with influenza and SARS-CoV-2, as they were free of charge, and later with pneumococcal disease, opting for a suboptimal scheme due to economic constraints that persisted as a barrier in a delayed social support.

This patient completed an intensive pulmonary rehabilitation program of 12 weeks and was discharged back to primary healthcare, where continuation of care was not possible due to a lack of resources, and self-instituted practices were discontinued due to poor motivation.

Pharmacological therapy can reduce COPD symptoms, reduce the frequency and severity of exacerbations, and improve health status and exercise tolerance. As an individual with COPD type E, with persistent blood eosinophil counts of more than 300 cells/μL, triple inhaled therapy of long-acting beta-agonist (LABA)+long-acting muscarinic antagonist (LAMA)+corticosteroid (ICS) is recommended, despite the increased risk of pneumonia due to ICS [1,2]. Long-term treatment with azithromycin, up to one year, reduces the risk of exacerbations in patients prone to or with severe exacerbations [1]. Azithromycin was not prescribed in this case but could have been considered. However, at baseline, this is a non-compliant patient, with a low understanding of his condition and how his behaviours influence his health status. Medication reconciliation and inhaler technique have been thoroughly reviewed and adjusted to his needs, but errors persist as therapeutic interventions increase their complexity, the latest with non-regular use of NIV and LTOT, leading to severe COPD exacerbations and emergency department recent admissions.

Depression is an important and underdiagnosed comorbidity in COPD [1]. In this case, depression was aggravated by the patient's social context, poor supportive network, and poverty. Despite the articulated work of the family medicine doctor and mental health nurse, pharmacology strategies were added and deemed insufficient.

Given the complex management of this case at a primary healthcare level, with continued aggravating status, we have signalled the palliative care team, aiming for a timely end-of-life intervention, effective symptom control, and a further holistic approach.

Overcoming therapeutic inertia as well as effectively articulating care between professionals is key in COPD, as opportunities for intervention are scarce in a fast-progressing disease. This case highlighted a non-compliance factor from the patient side, adding complexity to its management.

## Conclusions

A diagnosis of COPD should be considered in any patient who has dyspnea, chronic cough or sputum production, a history of recurrent lower respiratory tract infections, and/or a history of exposure to risk factors for the disease such as cigarette smoke.

Smoking cessation and pulmonary rehabilitation are essential, physical activity is recommended, and adequate vaccination has a major impact on the natural course of the disease. Personalized self-management education is pivotal.

The pharmacological treatment regimen should be individualized and guided by the severity of symptoms, risk of exacerbations, side effects, comorbidities, drug availability, and cost; pharmacological options often need to be considered throughout the progression of the disease, and patient response and preferences are privileged. The inhalation therapy needs to be reassessed in all appointments, accounting for symptoms, history of exacerbations, inhalation technique, and therapeutic compliance.

Concomitant chronic diseases occur frequently in COPD patients as was the case with our patient. These conditions should be actively sought and treated appropriately when present, as they influence health status, hospitalizations, and mortality.

In addition, all patients with advanced COPD should be considered for end-of-life and palliative care support, allowing patients and their families to make informed choices about future management.

This clinical case details how demanding a holistic approach can be to a patient with COPD in a primary healthcare setting. The progressive nature of this disease, with exacerbations, requires an assessment of its various aspects, in every encounter, demanding prompt and effective teamwork among levels of care.
